# Pneumonia-associated death in patients with dementia: A systematic review and meta-analysis

**DOI:** 10.1371/journal.pone.0213825

**Published:** 2019-03-14

**Authors:** Toshie Manabe, Yuji Fujikura, Katsuyoshi Mizukami, Hiroyasu Akatsu, Koichiro Kudo

**Affiliations:** 1 Department of Hygiene and Public Health, Teikyo University School of Medicine, Tokyo, Japan; 2 Department of Medical Risk Management and Infection Control, National Defense Medical College Hospital, Saitama, Japan; 3 Department of Internal Medicine, National Defense Medical College, Saitama, Japan; 4 Department of Social Health and Stress Management, Graduate School of Comprehensive Human Science, University of Tsukuba, Tsukuba, Japan; 5 Faculty of Health and Sport Sciences, University of Tsukuba, Tokyo, Japan; 6 Department of Community-Based Medicine, Nagoya City University Graduate School of Medicine, Nagoya, Japan; 7 Fukushimura Hospital, Toyohashi, Japan; 8 Waseda University Organization of Regional and Inter-Regional Studies, Tokyo, Japan; 9 Yurin Hospital, Tokyo, Japan; University of Malaya, MALAYSIA

## Abstract

**Background:**

Pneumonia is a serious disease associated with mortality among patients with dementia. However, the reported frequency of pneumonia as a cause of death in patients with dementia varies, the reason for which has not been fully elucidated.

**Methods:**

We conducted a systematic search in PubMed and the Cochrane Database of Systematic Reviews (inception to December 2016). Two authors independently determined the suitability of studies and potential bias and extracted the data. The primary outcome was frequency of pneumonia-associated death in patients with dementia. Stratified subgroup analysis was conducted among studies grouped according to type of mortality cause (immediate or underlying), information source of mortality cause (autopsy or death certificate), and study setting (clinic, hospital, or nursing home).

**Results:**

We included 7 studies reporting the cause of death among patients with dementia and 12 studies comparing the cause of death among patients with and without dementia. The frequency of pneumonia-associated death among 19 eligible studies was 29.69% (95% confidence interval [CI], 25.86–33.53). Those frequencies differed according to whether the source for information about cause of death was an autopsy confirmation (49.98%; 95% CI, 43.75–56.71) or death certificate (19.65%; 95% CI, 15.48–23.83) and according to whether the type of mortality cause was an indirect cause of death (13.96%; 95% CI, 9.42–18.51) or direct cause of death (44.45%; 95% CI, 29.81–50.10). The risk of pneumonia-associated death in patients with dementia was twice as high as among those without dementia (odds ratio, 2.15; 95% CI, 1.63–2.83; *p* < 0.001).

**Conclusion:**

The various frequencies of pneumonia-associated death in patients with dementia were associated with the information source, type of mortality cause, and study setting. Patients with dementia in the terminal stages urgently require careful clinical management of pneumonia, to maximize patient life expectancy and quality.

## Introduction

Pneumonia is a primary cause of hospitalization and mortality, especially for older adults [1,2]. With rapid growth of the older population, the importance of the clinical management of pneumonia is growing. The aging trend is accompanied by an increasing number of patients with dementia, which is becoming a major healthcare challenge [3]. Our previous study indicated that dementia was a risk factor for the occurrence of aspiration pneumonia in older adults [4]. Several studies have also reported that people with dementia tend to die more often from pneumonia [5–10]. A previous meta-analysis indicated that the odds of pneumonia-associated death were increased more than twofold for patients with dementia than for those without dementia [11]. However, the reported frequency of pneumonia-associated death among older adults with dementia varies, ranging from 12% to 70% [6–13]. Dementia covers a wide range of symptoms and encompasses a group of related neurodegenerative disorders. The various clinical factors relating to pneumonia-associated death are likely to coexist. Therefore, we hypothesized that the frequency of pneumonia-associated death differ depending on the methods used to obtain information about the cause of death (autopsy or death certificate), types of mortality cause (immediate or underlying cause), study settings, and the subtypes of dementia investigated.

The aims of the present study were to elucidate the frequency of pneumonia-associated death in older adults with dementia and how the frequency of pneumonia-associated death differ according to the data on cause of death (autopsy or death certificate). The results can contribute to the clinical management of patients with dementia in preventing pneumonia, to maximize life expectancy in these patients.

## Methods

This systematic review and meta-analysis was conducted according to the Preferred Reporting Items for Systematic Reviews and Meta-Analyses (PRISMA) statement and the statement by the Meta-analysis of Observational Studies in Epidemiology (MOOSE) group [14, 15]. A pre-defined protocol was not registered. Institutional review board approval and patient consent were not required because of the review nature of this study.

### Search strategy

Two investigators (TM and YF) independently searched for eligible studies in PubMed and the Cochrane Database of Systematic Reviews, published from database inception to December 2016. We used the following key words: “(dementia OR Alzheimer’s dementia OR Alzheimer’s disease OR Alzheimer disease OR dementia with Lewy bodies OR diffuse Lewy body disease OR vascular dementia OR frontotemporal dementia OR mixed-type of dementia) AND (pneumonia OR lower respiratory tract infection OR bronchopneumonia OR aspiration pneumonia OR nosocomial pneumonia OR community-acquired pneumonia OR hospital-acquired pneumonia OR nursing and healthcare-associated pneumonia OR ventilator-associated pneumonia) AND (mortality OR death OR comorbidity)”. The search was limited to studies written in English. The detailed search strategy is available in the supplementary appendix (S1 File). We also reviewed the reference lists of eligible studies using Google Scholar and performed a manual search to ensure that all appropriate studies were included.

### Eligibility criteria and outcome measures

Studies fulfilling the following selection criteria were included in the meta-analysis: (1) study design and language: randomized controlled trials, cohort studies, cross-sectional studies, and case series in English language; (2) population: adult patients with dementia or without dementia (as control patients); (3) primary outcome variables: the distributions of pneumonia-associated death. In the secondary outcome; (4) secondary outcome: the effect size on the odds ratio (OR) for pneumonia-associated mortality in patients with dementia was compared with patients without dementia. Studies were excluded based on the following criteria: (1) studies that only had abstracts; (2) studies where the outcome variable was not reported; and (3) studies that presented only an approximate frequency of pneumonia-associated death without the exact number of patients.

We conducted subgroup analysis among studies grouped according to the source of information about the cause of death, type of mortality cause, and study setting, to investigate pneumonia-associated death.

### Data extraction

Two reviewers extracted the data independently. Articles retrieved in the search were stored in a citation manager (EndNote X7; Thomson Reuters, New York, NY, USA). After removing redundant articles, titles and abstracts and then full-text articles were investigated. We extracted the following data: study design, study period, study site, study setting (clinic, hospital, or nursing home), inclusion/exclusion criteria of each study, information source of the cause of death (autopsy or death certificate), type of mortality cause (immediate or underlying), general patient background, and dementia type. Outcome variables were extracted into predesigned data collection forms. We verified data accuracy by comparing the collection forms of each investigator; any discrepancies were resolved through discussion together with three other authors (KM, HA and KK).

In previous meta-analyses, the underlying cause of death was defined as the disease, injury, or corresponding circumstance that initiated the chain of events (i.e., the intermediate cause of death) ultimately leading to death (7, 8, 9). The immediate cause of death was defined as the final disease, injury, or complication directly causing death (7, 8, 9).

### Data analysis

Throughout the meta-analysis, we calculated the prevalence of pneumonia-associated death or ORs with 95% confidence interval (CIs) using a random effects model, generic inverse variance method. To assess the prevalence of pneumonia-associated mortality among patients with dementia, the standard error was calculated using the Agresti–Coull method [16]. Heterogeneity among the original studies was evaluated using I^2^ statistics and classified as no heterogeneity (I^2^ = 0), low (≤ 25%), medium (25%–50%), and high (≥ 75%) [17]. Publication bias was examined using a funnel plot. For all analyses, significance levels were two-tailed, and *p* < 0.05 was considered significant. All statistical tests were performed using Review Manager (RevMan) ver. 5.3.5 (Cochrane Collaboration, Copenhagen, Denmark) [18].

## Results

### Study selection and characteristics

**Fig 1:**
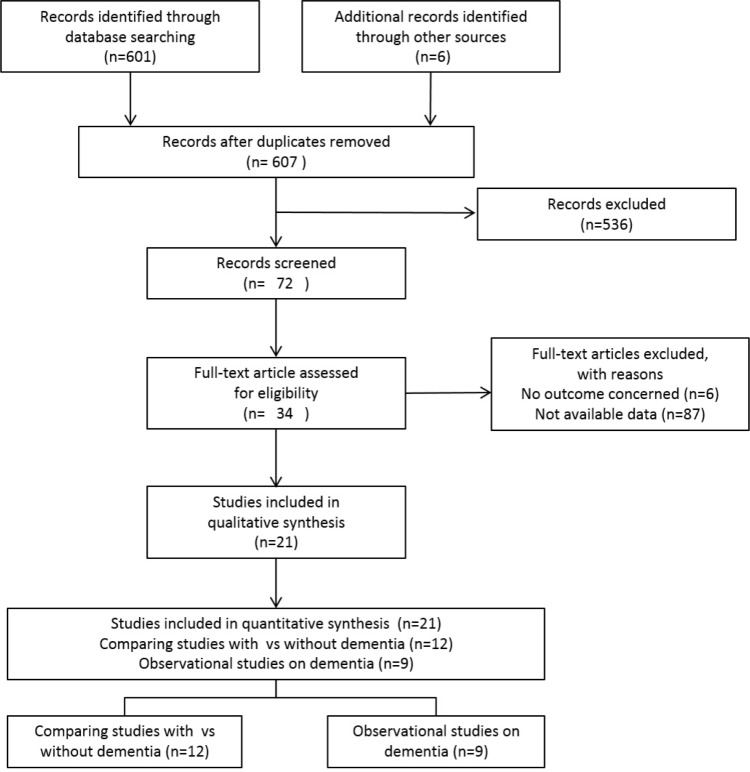
Systematic review flow diagram. n is the number of articles.

Of the 607 references screened, 7 studies [6, 9, 12, 19–22] reported the cause of death among patients with dementia; 12 studies [7, 8, 10, 13, 23–30] identified pneumonia-associated death in comparative studies reporting the cause of death among patients with dementia versus those without dementia (Fig 1).

In a total of 19 studies, the distribution of pneumonia-associated death was analysed in 79,956 patients with dementia. Among the 19 studies, autopsy-confirmed cause of death was used in all hospital-based studies [6, 8–10, 12, 19, 26] except one [27]; 8 population-based studies [7, 13, 22, 23, 25, 28–30], 2 studies in nursing homes [20, 21] and 1 study in a clinic [24] used death certificates as the source for cause of death (Table 1). In the 12 comparative studies, 40,039 patients with dementia and 332,456 without dementia were compared for pneumonia-associated death. The most common type of dementia was Alzheimer disease (AD) and four studies included only patients with AD [19, 21, 24, 29].

**Table 1:** Baseline characteristics of included studies comparing pneumonia-associated mortality in patients with and without dementia.

Study, year of publication	country	Study setting	Study design	Resource that provided information for cause of death	Cause of death; (underlying or immediate)	Sample size	Mean age–yr ±SD	Gender–Female, n (%)	Types of dementia among patients with dementia- (%)
**Study examining cause of death on dementia**									
Burns, 1990 [19]	UK	Hospital	longitudinal	Autopsy and death certificate	-	53	80.4 (range, 56–99)	79	AD (100.0)
Fu, 2004 [6]	USA	Hospital	retrospective	Autopsy	-	52	77.6 ± 10.8	48.1	AD (55.8), AD and DLBD (3.8), FTD (9.4), VaD (5.7)
Wachterman, 2008 [20]	USA	Nursing home	cohort	Death certificate	immediate	165	86.5 ± 7.2		End-stage dementia
Brunnstrom, 2009 [9]	Sweden	Hospital	retrospective	Autopsy	underlying & immediate	524	78.6 ± 9.1	55.3	AD (42), VaD (23.7), AD and VaD (21.6)
Bosek, 2013 [21]	USA	Nursing home	retrospective	Clinical diagnosis		57	-		AD (100)
Manabe, 2015 [12]	Japan	Hospital	retrospective	Autopsy	immediate	157	84.5 ± 8.5	61.3	AD (40.1), DLB (26.8), VaD (33.1)
Vorst,2016 [22]	Netherlands	Population based (day clinic or inpatient)	cohort	Death certificate	underlying	39164	81.4 ± 7.0	61.3	AD (62.4), VaD (12.5), Others (25.1)
**Comparative study on patients with versus without dementia**									
Kukull, 1994 [7]	USA	Population	retrospective	Death certificate	underlying	87 vs. 17	82.5 ± 5.0 (probable AD)	54.0	AD (11.5), probable AD (63.2), others (25.3)
Morgan and Clarke, 1995 [23]	UK	Population	prospective cohort	Death certificate	principal	64 vs. 448	≥75 y, 82.9%	65.7	-
Beard, 1996 [24]	USA	clinic	case-control	Death certificate	underlying & immediate	917 vs. 703	-	-	AD (100)
Kammoun, 2000 [8]	Switzerland	Hospital	retrospective	Autopsy	immediate	120 vs. 222	85.0 ± 6.9		AD (76.7), VaD 15.5), MTD (9.6), others
Tschanz, 2004 [25]	Sweden	Population	county study	Death certificate	-	291 vs. 947	83.3 ± 7.0	64.0	AD (57.7), VaD (15.5), MTD (9.6), others
Attems, 2005 [26]	Austria	Hospital	retrospective	Autopsy	-	176 vs. 132	83.5 ± 8.6	58.1	AD, VaD, MTD, others
Laditka, 2005 [27]	USA	Hospital	retrospective	Death certificate	-	36887 vs. 327425	-	-	-
Chamandy and Wolfson, 2005 [28]	Canada	Population	cohort	Death certificate	underlying	754 vs. 618	87.6 ± 7.26	68.2	AD, VaD, MTD, others
Ganguli, 2005 [29]	USA	population	cohort	Death certificate	underlying	236 vs. 546	73.4 ± 5.9	57.8	AD (100)
Andersen, 2010 [30]	Denmark	Population	cohort	Death certificate		286 vs. 884	81.2 ± 3.8	64.3	AD (67.4)VaD (19.1), MTD and others,
Todd, 2013 [13]	USA	Population	cohort	Death certificate	underlying	85 vs. 52	78.6 ± 7.5	68.3	-
Magaki, 2014 [10]	USA	Hospital	retrospective	Autopsy	immediate	45 vs. 124	78.5 ± 11.5	47.7	AD (80.3), FTD (6.4), DLBD (3.2), others

Abbreviations: AD, Alzheimer disease; DLBD, diffuse Lewy body disease; FTD, frontotemporal dementia; VaD, vascular dementia, MTD, mixed-type dementia. Sample size among comparative studies presented as patients with versus patients without dementia.

### Prevalence of pneumonia-associated death among patients with dementia

**Fig 2:**
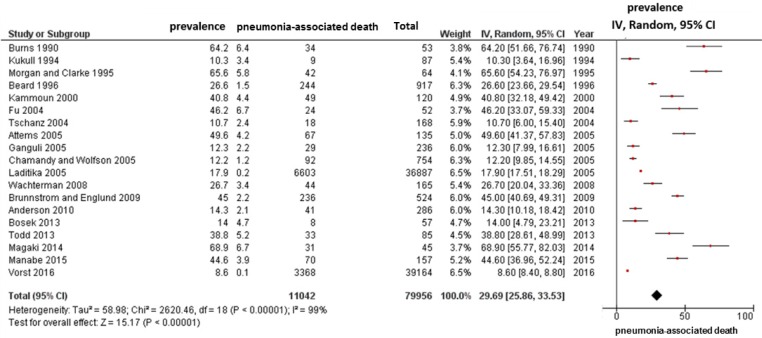
Meta-analysis for frequencies of pneumonia-associated death in patients with dementia. Data for all 19 studies on patients with dementia. Cumulative meta-analysis using a random effects model for frequency of pneumonia-associated death is shown, by study. Squares represent 95% confidence intervals (CIs). Diamonds at the bottom of the figure show 95% CI range of the overall estimates.

In all 19 studies, we identified 79,956 patients with dementia and estimated the prevalence of pneumonia-associated death. The result indicated that 29.69% (95% CI, 25.86–33.53; I^2^ = 99%; *p* for heterogeneity < 0.001) of patients with dementia died owing to pneumonia (Fig 2).

**Fig 3:**
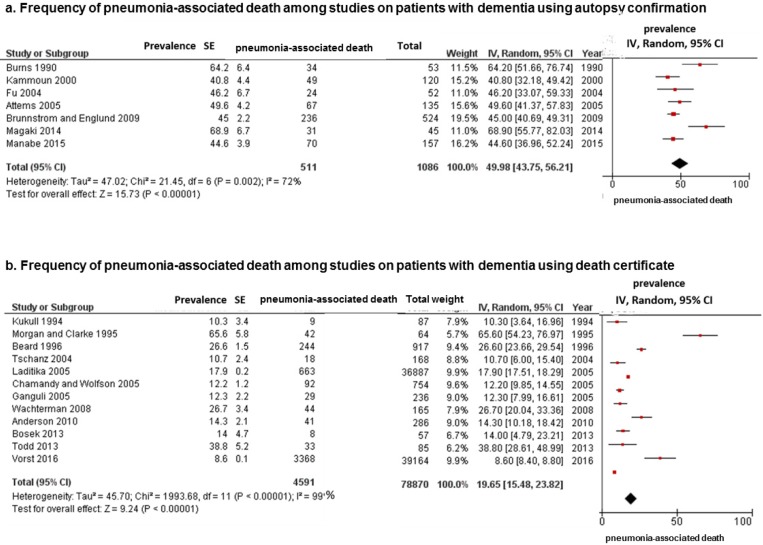
Meta-analysis for frequencies of pneumonia-associated death in patients with dementia according, to information source for the cause of death. (a) Data from 7 studies using autopsy reports. (b) Data from 12 studies using death certificates. Cumulative meta-analysis using a random effects model for frequency of pneumonia-associated death is shown, by study. Squares represent 95% confidence intervals (CIs). Diamonds at the bottom of the figure show 95% CI range of the overall estimates.

In the subgroup analysis according to information source for the cause of death (autopsy or death certificate), the estimated frequency of pneumonia-associated death in studies using autopsy confirmation was 49.98% (95% CI, 43.75–56.21; I^2^ = 72%; *p* for heterogeneity = 0.002) (Fig 3A) whereas that in studies using death certificates was 19.65% (95% CI, 15.48–23.82; I^2^ = 99%; *p* for heterogeneity < 0.001) (Fig 3B).

**Fig 4:**
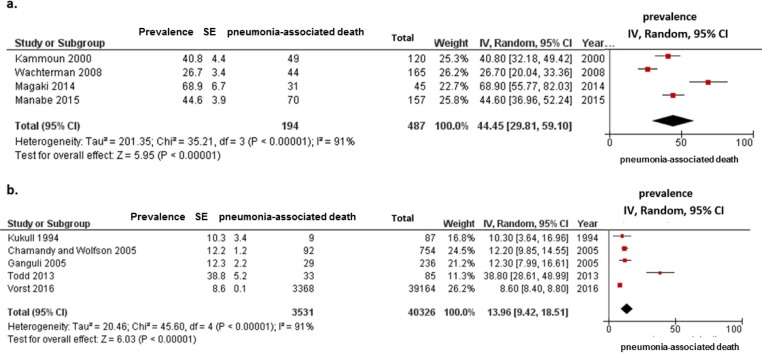
Meta-analysis for frequencies of pneumonia-associated death in patients with dementia, according to type of mortality cause. (a) Data from four studies that reported pneumonia as an immediate cause of death. (b) Data from five studies that reported pneumonia as a underlying cause of death. Cumulative meta-analysis using a random effects model for frequency of pneumonia-associated death is shown, by study. Squares represent 95% confidence intervals (CIs). Diamonds at the bottom of the figure show 95% CI range of the overall estimates.

In the second subgroup analysis according to type of mortality cause (immediate or underlying), the estimated frequency of pneumonia-associated death as immediate cause was 44.45% (95% CI, 29.81–59.10; I^2^ = 91%; *p* for heterogeneity < 0.001) (Fig 4A) whereas the estimated frequency of those as underlying cause was 13.51% (95% CI, 9.42–18.51; I^2^ = 91%; *p* for heterogeneity < 0.001) (Fig 4B).

**Fig 5:**
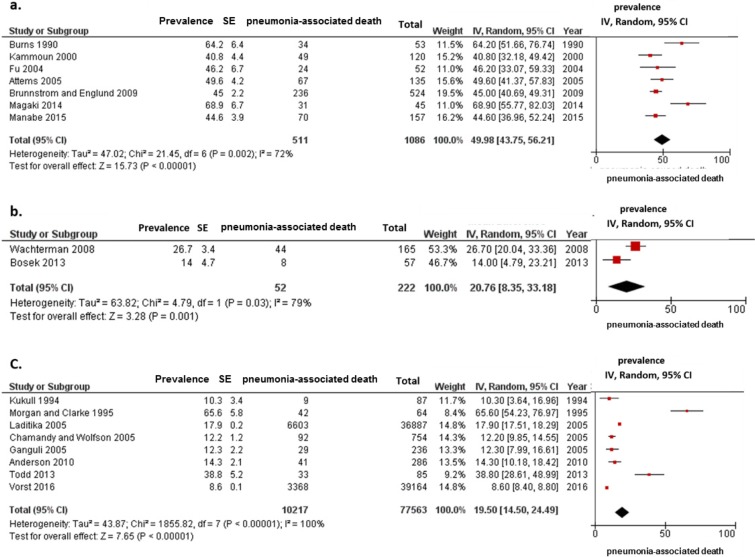
Meta-analysis for frequencies of pneumonia-associated death in patients with dementia according to study settings. (a) Data of seven hospital-based studies. (b) Data of two nursing home-based studies. (c) Data of eight population-based studies. Cumulative meta-analysis using a random effects model for frequency of pneumonia-associated death is shown, by study. Squares represent 95% confidence intervals (CIs). Diamonds at the bottom of the figure show 95% CI range of the overall estimates.

In the final subgroup analysis according to study setting (clinic, hospital, or nursing home), the frequency of pneumonia-associated death in hospital-based studies was the same as that in studies using autopsy confirmation (Figs 3A and 5A) and higher than the frequency in nursing home-based studies (20.76%; 95% CI, 8.35–33.18; I^2^ = 79%; *p* for heterogeneity = 0.001) (Fig 5B) and population-based studies (19.50%; 95% CI, 14.50–24.49; I^2^ = 100%; *p* for heterogeneity < 0.001) (Fig 5C).

### Risk of pneumonia as a cause of death in patients with and without dementia

**Fig 6:**
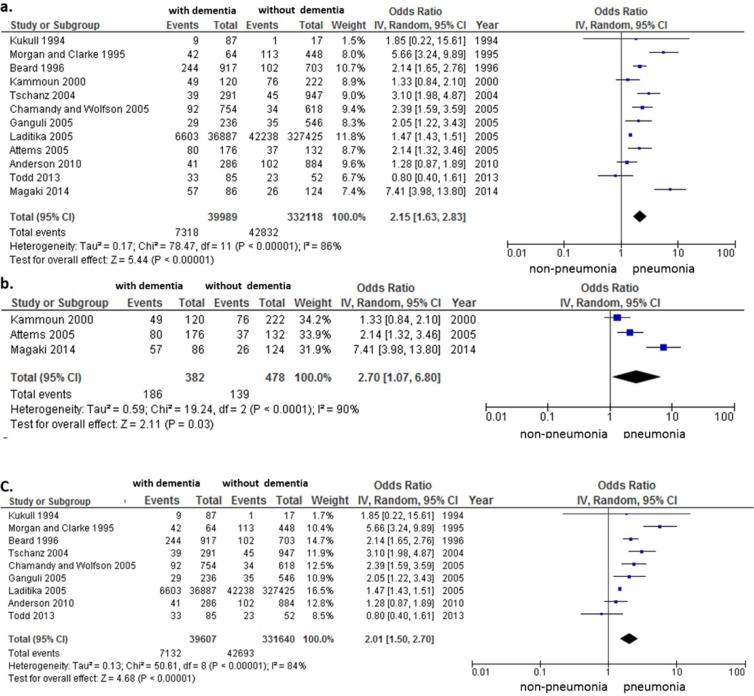
Risk of pneumonia-associated mortality in patients with and without dementia. (a) Data for 12 studies that compared patients with versus those without dementia. (b) Data from 3 studies that examined pneumonia-associated death using autopsy reports. (c) Data from 9 studies that examined pneumonia-associated death using death certificates. Cumulative meta-analysis using a random effects model with odds ratios (ORs) shown by study. Squares represent 95% confidence intervals (CIs). Diamonds at the bottom of the figure show 95% CI range of the overall estimates.

Among the 12 comparison studies that reported pneumonia-associated death among patients with dementia versus those without dementia, cumulative analysis showed a significant risk of pneumonia as a cause of death in patients with dementia, as compared with those who did not have dementia (OR, 2.15; 95% CI, 1.63–2.83; *p* < 0.001) (Fig 6A). However, the results among studies of autopsy-confirmed patients were higher (OR, 2.70; 95% CI, 1.07–6.80; *p* = 0.03) (Fig 6B) than those of studies that obtained the cause of death from a death certificate (OR, 2.01; 95% CI, 1.50–2.70; *p* < 0.001) (Fig 6C).

## Discussion

The present systematic review and meta-analysis revealed that the frequency of pneumonia-associated death in patients with dementia varied according to the information source, type of mortality cause, and study setting. The frequency of pneumonia-associated death in patients with dementia was 49.98% if the source of information for cause of death was autopsy confirmation. This frequency was 2.5 times higher than that in studies using a death certificate (19.65%) as well as nursing home-based (20.76%) and population-based (19.50%) studies. The frequency of pneumonia as an immediate cause of death (44.45%) was three times higher than as an underlying cause of death (13.51%). Patients with dementia had a two-times greater risk of death owing to pneumonia compared with patients who did not have dementia.

With rapid growth of the older population, pneumonia has become one of the most important infectious diseases in terms of frequency, disease prognosis, and impact on society. Older adults with dementia in particular are more likely to die from pneumonia than those without dementia [6–9, 11]. Although the number of older adults facing dementia has been increasing globally, the current situation is that the clinical evidence for managing patients with dementia remains insufficient. Therefore, we could only evaluate 19 studies in the present study, to confirm the risk of pneumonia-associated death among older adults with dementia. Our results indicated the risk of pneumonia-associated death was increased more than twofold in patients with dementia (OR, 2.15) (Fig 6). These results indicated that patients with dementia may have weaker defence mechanisms for overcoming respiratory tract infections than those without dementia. Previous studies have revealed an association between respiratory function and cognition, which is impaired in dementia [31–33]. The deterioration of respiratory function may also influence the reduced lifespan once patients with dementia develop pneumonia. However, the reported frequency of pneumonia-associated death varies among patients with dementia [6–13]. In addition, the ORs of pneumonia-associated death differed according to whether the source of information about the cause of death was a death certificate or an autopsy report (Fig 6B and 6C). It is important to investigate these differences in frequency of pneumonia-associated deaths in daily clinical practice for patients with dementia, among which the number of such deaths is increasing rapidly, with the global aging population.

Among all eligible studies including patients with dementia, the frequency of pneumonia as a cause of death was approximately 20% in studies using death certificates (Fig 3C); however, this frequency was approximately 50% in studies using autopsy reports in hospital settings (Fig 3B). This high frequency of pneumonia cause of death in patients with dementia among studies using death certificates was similar to the pneumonia-associated deaths in studies reporting an immediate cause of death (Fig 4A) as well as in population-based (Fig 5C) and nursing home-based (Fig 5B) studies. Although comorbid conditions among patients in the present study could not be examined, our results suggest that pneumonia has a strong and direct impact on mortality in older adults with dementia, regardless of whether they have other, possibly fatal underlying conditions. In fact, studies using autopsy reports presented the immediate cause of death, which is defined as the final disease, injury, or complication directly causing death [9]. However, studies using a death certificate presented the underlying cause of death, which is the disease, injury, or corresponding circumstance that initiated a chain of events ultimately leading to death [9]. In our previous study, pneumonia-associated deaths among patients with dementia accounted for 35.0% to 44.6% of deaths in all patients, for the underlying and immediate causes of death, respectively [12]. The clinical diagnosis of pneumonia in older adults is difficult and often delayed because of atypical or paucisymptomatic presentations including the absence of fever, paucity or absence of cough, changes in mental status (delirium), and poorly contributive physical examination [1, 34]. Silent pneumonia may have a greater impact as the direct cause of death than clinical presentation among older adults, especially those with advanced stages of dementia in the hospital setting. The important physical changes associated with aging include decreases in the elastic recoil of the lungs, compliance of the chest wall, and strength of the respiratory muscles [24]. Parkinsonism during the course of Alzheimer’s disease (AD) [35] and sequelae of cerebral vascular disease also contribute to decreasing respiratory muscle function [36, 37]. A previous study suggested that weakness of the extremities in patients with dementia with Lewy bodies (DLB) may be associated with low respiratory function [38]. Thus, such decreased respiratory functions have important consequences on the functional reserve of older adults with dementia, leading to a further decreased ability to cope with reduced lung compliance and increased airway resistance. Therefore, once these patients develop a lower respiratory tract infection, they can easily develop recurrent pneumonia, which can lead to pneumonia-caused mortality.

Although we were unable to evaluate the status of dysphagia among patients in the present study, most were in the terminal stage of illness and their cognitive impairments might have been severe. One study reported that patients with dementia (mean age 86 years) inevitably develop dysphagia and have a high risk of aspiration pneumonia, which is related to hospital-based mortality [39]. A previous meta-analysis suggested that the prevalence of swallowing difficulties ranges from 13% to 57% in different types of dementia, and the prevalence of swallowing difficulties in patients with DLB is higher than in those with AD. Owing to limited data availability for the present meta-analysis, the most common form of dementia among the included patients was AD (Table 1). Although we were unable to conduct an evaluation according to the different forms of dementia, dysphagia, which may lead to the development of fatal pneumonia, may differ in different forms of dementia associated with lesions in diffuse areas of the brain, which result in disorders of cognition and deterioration in oral, pharyngeal, and laryngeal functioning [40]. Further studies are needed to clarify this question.

The present meta-analysis was limited to the evaluation of published data. The eligible studies in this meta-analysis included population-based studies and hospital, clinic, and nursing home-based studies. The various study settings, general clinical conditions, comorbidities, and disease severity among patients in the present study varied widely. However, dementia covers a wide range of symptoms and encompasses a group of related neurodegenerative disorders. As the nature of disease among study participants as well as the nature of meta-analysis precludes the use of individual patient data, the heterogeneity among patients in each study could not be determined. There was also the possibility of different criteria used to determine pneumonia as the cause of death among studies. The present study findings warrant the further investigation among patients with the same subtypes of dementia as well as the same underlying clinical conditions.

## Conclusion

We found that approximately 50% of patients with dementia in the hospital setting died owing pneumonia, according to cause of death confirmed by autopsy. This frequency may be much higher than clinician’s expectations. Our results indicated that clinicians must pay careful attention in cases of pneumonia among patients with dementia in the terminal stages of illness, to maximize patients’ life expectancy and quality.

## Supporting information

S1 File Detailed search strategy (Pubmed). (DOCX)

S2 File PRISMA checklist. (DOCX)
