# Supplementary material for: Pneumonia-associated death in patients with dementia: A systematic review and meta-analysis
Source: PLoS One. 2019 Mar 14;14(3):e0213825. doi: 10.1371/journal.pone.0213825 (PMC6417730; doi:10.1371/journal.pone.0213825)
Supplement: S1 File — (DOCX) [file pone.0213825.s001.docx]

**S1 Appendix**

**Detailed search strategy (Pubmed)**

((“pneumonia”[MeSH Terms] OR "pneumonia"[All Fields]) OR “lower respiratory tract infection”[All Fields] OR (“bronchopneumonia”[MeSH Terms] OR “bronchopneumonia”[All Fields]) OR “aspiration pneumonia”[All Fields] OR “nosocomial pneumonia”[All Fields] OR “community-acquired pneumonia”[All Fields] OR “hospital-acquired pneumonia”[All Fields] OR “nursing and healthcare-associated pneumonia”[All Fields] OR “ventilator-associated pneumonia” [All Fields]) AND (“mortality”[All Fields] OR “death” [All Fields] OR “cause of death”[All Fields] OR “cause of mortality”[All Fields] OR “comorbidity”[All Fields]) AND (“dementia”[MeSH Terms] OR "dementia"[All Fields]) OR (“Alzheimer disease”[MeSH Terms] OR "Alzheimer disease"[All Fields] OR “Alzheimer’s disease” [All Fields]) OR (“dementia with Lewy bodies[All Fields]” OR “diffuse Lewy body disease”[All Fields] OR “Lewy body disease” [MeSH Terms]”) OR “vascular dementia”[All Fields] OR “frontotemporal dementia”[All Fields] OR “mixed-type of dementia”[All Fields] OR “demented”[All Fields]).
